# Alveolar Soft Part Sarcoma of the Tongue in an Adolescent

**DOI:** 10.7759/cureus.11506

**Published:** 2020-11-16

**Authors:** Amaranto Suárez, Kelly Paz, Mario Melo-Uribe, Andrey Moreno, Alejandra Calderon

**Affiliations:** 1 Pediatric Oncology, Instituto Nacional de Cancerología, Bogotá, COL; 2 Pathology, Instituto Nacional de Cancerología, Bogotá, COL; 3 Head and Neck Surgery, Instituto Nacional de Cancerología, Bogotá, COL

**Keywords:** tongue, children, non-rhabdomyosarcoma soft tissue sarcoma, alveolar soft part sarcoma

## Abstract

Alveolar soft part sarcoma is a rare malignant soft tissue neoplasm of uncertain histogenesis and aggressive clinical behavior. Alveolar soft part sarcoma arises in the head and neck in 27% of cases, with 25% of head and neck cases occurring in the tongue. Herein the case of a pediatric patient diagnosed with alveolar soft tissue sarcoma of the tongue, who received surgical treatment with total resection of the lesion and chemotherapy without radiotherapy, is presented. To date, the patient is in remission of the disease.

## Introduction

Alveolar soft part sarcoma (ASPS) is a rare malignant neoplasm of uncertain histogenesis, first described in 1952 by Christopherson [1,2,3]. It represents between 0.4% and 1.0% of all soft tissue sarcomas and occurs in 5% of all non-rhabdomyosarcoma soft tissue sarcomas in pediatrics [4,5]. It predominantly affects adolescents and young adults between 15 and 35 years of age with a predominance in women, with an H:M ratio of 1:1.4 [6,7].

In adults, the most common location is the lower extremities' deep soft tissues (thighs and buttocks) in contrasts with the presentation in children and adolescents where there is a predilection for the location in the head and neck, in particular, the tongue (where it occurs in approximately 25% of cases) and the orbit [8,9,10].

Clinically indolent, it presents as a painless, slow-growing mass with great vascularity; the limited symptoms generate delays in diagnosis, which could explain that 80% of adult patients and 37% of pediatric patients present metastases at diagnosis [11]. The disease has a survival rate of 77% at two years, 60% at five years, 38% at 10 years, and 15% at 20 years [11]. In its localized form of presentation, five-year survival is 71%; in metastatic disease, it is only 20% [11].

Alveolar soft part sarcoma of the tongue is a highly vascular tumor; diagnostic studies such as ultrasound can misdiagnose this tumor and be mistaken for benign lesions; so magnetic resonance imaging is the study of choice [10,11]. The initial diagnostic suspicion of alveolar soft part sarcoma of the tongue is complicated, causing delays in referral to specialized centers where the diagnosis is established [10,11].

## Case presentation

**Figure 1:**
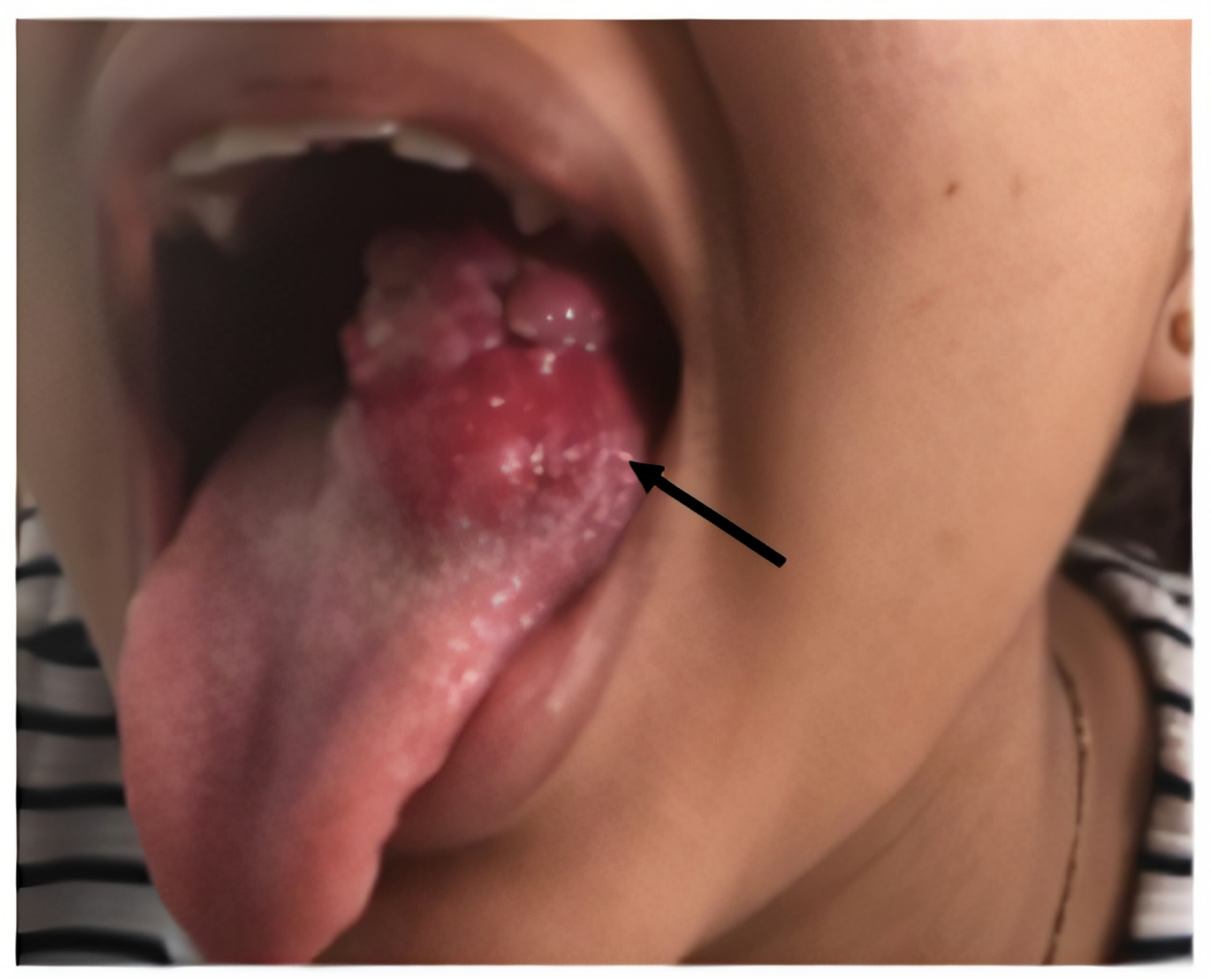
Tongue mass. Non-ulcerated, painless, and lobulated mass on the left lateral ventral surface of the tongue,

A 13-year-old adolescent woman presented with a 14-month history of a non-ulcerated, painless, and lobulated mass on the left lateral ventral surface of the tongue, slow-growing, without other associated symptoms (Figure 1).

**Figure 2:**
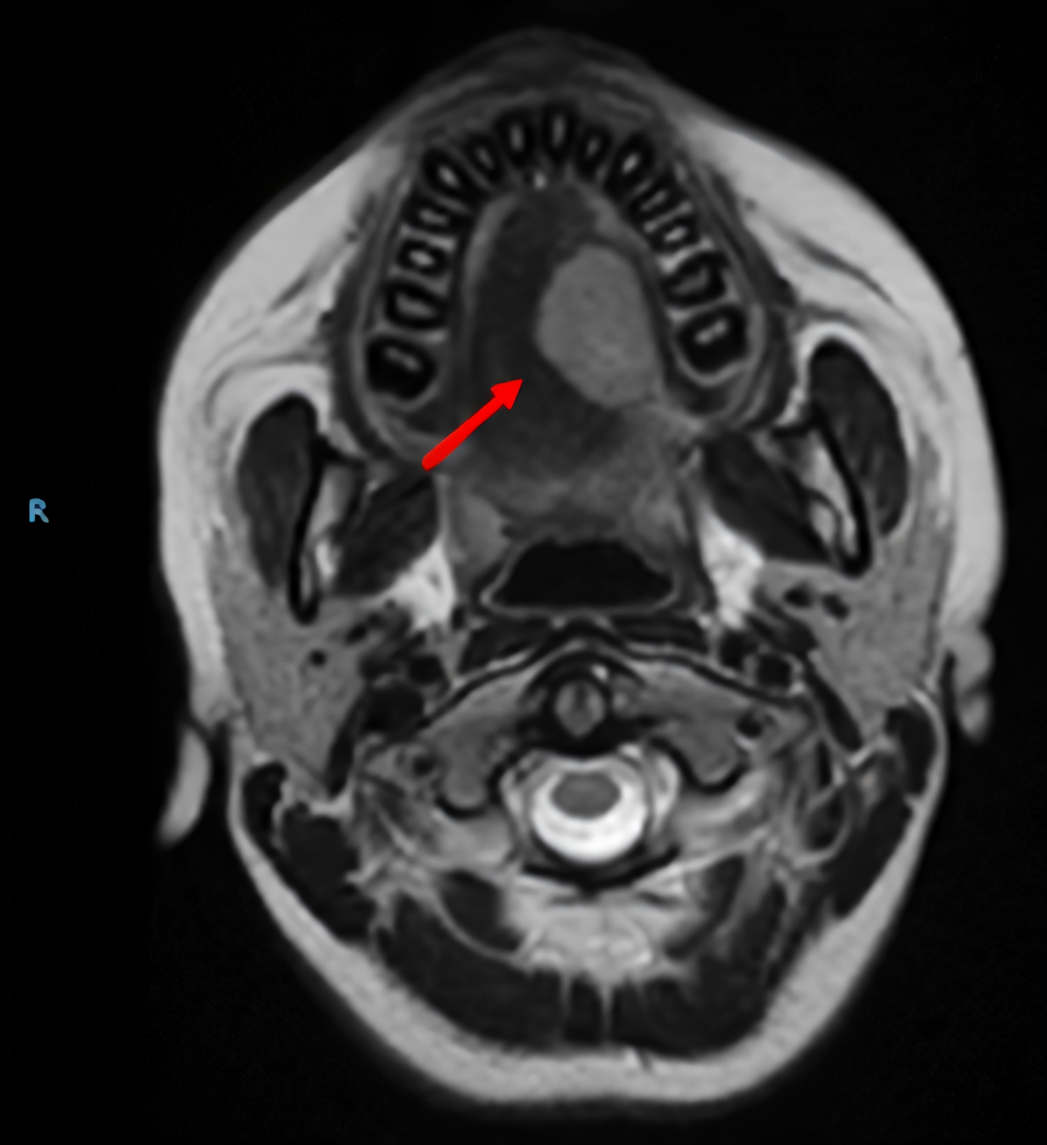
Magnetic resonance imaging of the face and neck. Involvement of the mucosa and the superior longitudinal, vertical, and transverse muscles of the lateral dorsum of the left hemilanguage was observed by a mass of well-defined and regular contours with increased intensity of signal in the T2-weighted sequences, with intense and homogeneous enhancement with the contrast medium, with measurements of 21 mm x 26 mm x 21 mm.

A nuclear magnetic resonance imaging of the face and neck was performed, which showed a mass of well-defined and regular contours in the superior longitudinal, vertical, and transverse muscles of the lateral dorsum of the left hemitongue (Figure 2).

**Figure 3:**
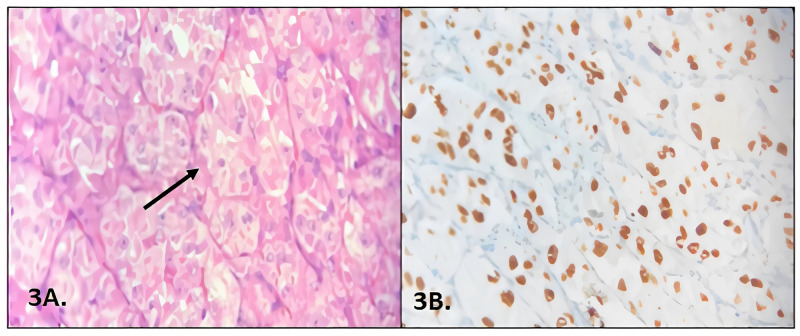
Pathology and immunohistochemistry. 3A: Lesion of well-defined nests of polygonal cells, eosinophilic granular cytoplasm, surrounded by thin septa fibrous, giving the appearance of alveoli formation, compatible with alveolar soft tissue sarcoma. 3B: Transcription factor E3 (TFE3) positive.

With a probable diagnosis of the tongue's hemangioma, resection was performed in a center outside ours. The pathology report showed a lesion compatible with an alveolar soft part sarcoma with one of the resection edges in contact with the tumor (Figure 3).

Taking into account the pathology report, upon admission to our institution, a second surgery was proposed to widen the surgical margins and lymph node dissection. Considering the presence of one of the edges in contact with the tumor and the initial non-oncological resection, the tumor was staged as Group-III of the Intergroup Rhabdomyosarcoma Study Group (IRSG) staging system [12].

The initiation of chemotherapy was indicated; three cycles of ifosfamide and doxorubicin were administered every 21 days. Subsequently, surgical resection was performed to widen the edges. The pathology is reported without residual tumor, with negative resection borders.

**Figure 4:**
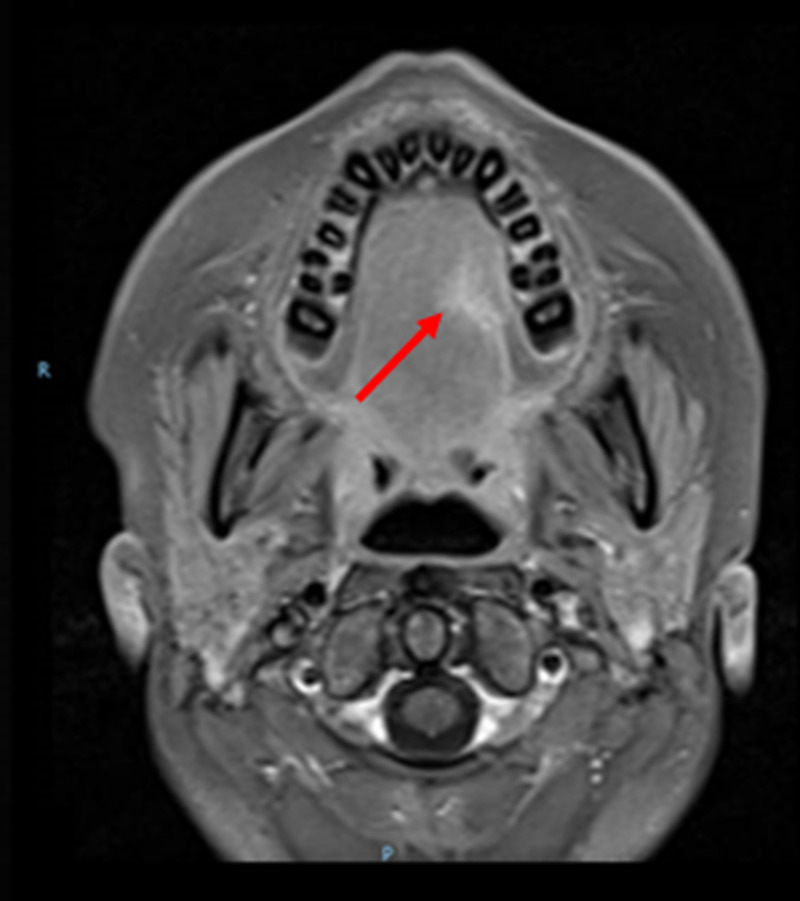
Post-treatment MRI. Post-treatment MRI shows the scar where the tumor was, with no signs of tumor relapse.

Three cycles of adjuvant chemotherapy were applied with the same agents, without radiotherapy. To date, the patient has completed treatment and is under follow-up, disease-free, and asymptomatic. Post-treatment MRI shows the scar where the tumor was, with no signs of tumor relapse (Figure 4). 

## Discussion

Soft part sarcoma is a rare neoplasm in pediatrics, accounting for 5% of all cases of non-rhabdomyosarcoma soft part sarcomas in pediatrics [4]. Despite being an indolent growth neoplasm, alveolar soft part sarcoma has a high propensity to metastasize, especially to the lung in up to 42%-65% of cases, with a metastatic presentation to the bones and brain being less common, even in early stages of the disease [11,12].

The five-year survival rate for alveolar soft tissue sarcoma in children, adolescents, and adults younger than 25 years is 83% [13]. However, due to the proclivity for late metastases, survival rates may decrease [13]. In the previously illustrated case, despite the time elapsed between the biopsy and the moment when negative resection margins were achieved, the patient did not present metastatic involvement.

In contrast to what was reported by Shelke et al. [11], who reported that the most frequent location of lingual alveolar sarcoma is the base of the tongue (18%), followed by 13% on the back, 6% on the ventral surface, and 3% on the left lateral back [11], the patient presented the tumor on the ventral and left lateral surface of the tongue.

The clinical course was indolent as described in the literature as a hemangioma of the tongue for which a surgery limited to tumor resection with a positive border was performed, which led to an enlargement of the margins and lymph node dissection. In the presence of a non-metastatic tumor and in a location where radical resections leave significant morbidity in the soft tissues of the neck and long-term cosmetic and psychological side effects, conservative management was carried out with neoadjuvant, adjuvant chemotherapy, and surgery. At the end of treatment, the patient is in remission, free of signs of tumor recurrence or relapse.

## Conclusions

It will always be important to take a detailed clinical history to reach the correct diagnosis of the soft part sarcoma of the tongue. A wrong diagnosis can lead to delay in the initiation of adequate treatment of the patient. The presentation of the disease in children, adolescents, and young adults makes us reflect on the need to form multidisciplinary groups with the active participation of adult clinical oncologists, head and neck surgeons, pathologists, radiation therapists, and rehabilitators.
